# A new humanized antibody is effective against pathogenic fungi in vitro

**DOI:** 10.1038/s41598-021-98659-5

**Published:** 2021-09-30

**Authors:** Tomas Di Mambro, Tania Vanzolini, Pierpaolo Bruscolini, Sergio Perez-Gaviro, Emanuele Marra, Giuseppe Roscilli, Marzia Bianchi, Alessandra Fraternale, Giuditta Fiorella Schiavano, Barbara Canonico, Mauro Magnani

**Affiliations:** 1 Department of Biomolecular Sciences, University of Urbino “Carlo Bo”, 61029 Urbino, Italy; 2 Instituto de Biocomputación y Física de Sistemas Complejos (BIFI), Universidad de Zaragoza, 50018 Zaragoza, Spain; 3 Departamento de Física Teórica, Universidad de Zaragoza, 50009 Zaragoza, Spain; 4 Centro Universitario de la Defensa, 50090 Zaragoza, Spain; 5 Takis S.R.L, Via di Castel Romano 100, 00128 Rome, Italy; 6 Department of Humanities, University of Urbino “Carlo Bo”, 61029 Urbino, Italy; 7 Diatheva S.R.L, Via Sant’Anna 131/135, 61030 Cartoceto, Italy

**Keywords:** Biologics, Bioinformatics, Immunological techniques, Antibody generation, Antibody isolation and purification, ELISA, Immunohistochemistry, Chromatography, Electrophoresis, Protein purification, Microbiology techniques, Fungal infection

## Abstract

Invasive fungal infections mainly affect patients undergoing transplantation, surgery, neoplastic disease, immunocompromised subjects and premature infants, and cause over 1.5 million deaths every year. The most common fungi isolated in invasive diseases are *Candida *spp*., Cryptococcus *spp*., *and* Aspergillus *spp. and even if four classes of antifungals are available (Azoles, Echinocandins, Polyenes and Pyrimidine analogues), the side effects of drugs and fungal acquired and innate resistance represent the major hurdles to be overcome. Monoclonal antibodies are powerful tools currently used as diagnostic and therapeutic agents in different clinical contexts but not yet developed for the treatment of invasive fungal infections. In this paper we report the development of the first humanized monoclonal antibody specific for β-1,3 glucans, a vital component of several pathogenic fungi. H5K1 has been tested on *C*. *auris,* one of the most urgent threats and resulted efficient both alone and in combination with Caspofungin and Amphotericin B showing an enhancement effect. Our results support further preclinical and clinical developments for the use of H5K1 in the treatment of patients in need.

## Introduction

More than 300 million people have serious fungal diseases and there are over 1.5 million deaths every year. Pathogenic fungi cause life-threatening infections such as fungaemia, pneumonia, chronic pulmonary aspergillosis, bronchopulmonary aspergillosis and cryptococcosis^1,2^. These pathologies affect mainly patients undergoing transplantation, surgery and neoplastic disease, immunocompromised subjects and premature infants. Annually are reported about 3,000,000 cases of chronic pulmonary aspergillosis, 223,100 cases of cryptococcal meningitis complicating HIV/AIDs, 700,000 cases of invasive candidiasis, 500,000 cases of *Pneumocystis jirovecii* pneumonia, 250,000 cases of invasive aspergillosis, 100,000 cases of disseminated histoplasmosis, over 10,000,000 cases of fungal asthma^3^. Of the existing five million fungal species (spp.), only 300 are considered dangerous for humans, and ~ 10% of them are recurrent. The most common fungi isolated in invasive diseases are *Candida* spp*.*, *Cryptococcus* spp., and *Aspergillus* spp. The mortality rate for invasive candidiasis is about 40%^4^, from 20 to 30% for cryptococcosis^5^ and 20% for aspergillosis. These data are referred to wealthy countries with a fully functional healthcare, while where resources are limited the death rate surpasses 50%^6^.

Candidiasis is the second most frequent fungal infection^7^. *C. albicans* is the most prevalent specie but the number of infections caused by non-*albicans Candida* species (NACs) is increasing. Moreover, the massive use of antifungal drugs has determined the selection of species with an innate resistance or higher tolerance. Together, *C. albicans*, *krusei*, *parapsilosis*, *glabrata* and *tropicalis* represents the 80% of the total cases of infections^8^ and 49.5% of them are caused by NACs. The different geographical diffusion determines not just the prevalence of one specie over the others but also the influences that it therefore receives, the different virulence, susceptibility, resistance, and risk factors. In line with these considerations the mortality is still high because the detection methods are often not species-specific, and the diagnoses are delayed as well as adequate antifungal therapies^7,9–11^.

In addition to all these complications, the discovery of new frightening fungal species makes the race for drugs more urgent. This is the case of *C. auris* which appeared for the first time in 2009^12^ and has spread rapidly all over the world. It commonly presents multidrug resistance to every class of antifungal drugs. The Minimum Inhibitory Concentration (MIC) of different strains for fluconazole range from 32 to ≥ 64 mg/l while for voriconazole it is 16 mg/l. Around 30% of the strains have a low susceptibility to Amphotericin B (MIC ≥ 2 mg/l) and recent studies have confirmed an increasing resistance to echinocandins (MIC ≥ 8 mg/l)^13,14^. *C. auris* is tolerant to high salt concentrations (where it tends to assume a rudimental pseudo-hyphal form) and to high temperatures (42 °C)^15^. *C. auris* can adhere to biotic and abiotic surfaces and colonize them for weeks and months becoming a very serious problem for the invasive devices used in the hospitals^13^. Part of its danger also comes from biofilm formation and from the production of phospholipases and proteinases^16^. This profile is probably not complete, but it does provide a reasonable explanation for 60% mortality^13^.

Facing these alarming numbers, more and more efforts have been spent in finding new antifungal drugs or in improving those already on the market. Yet, nowadays there are still just four leading classes of antifungal drugs: polyenes, pyrimidine analogues, azoles and echinocandins^17–20^. The lack of new therapeutic agents is mostly due to the large hurdles to overcome.

Toxicity represents the first issue of current and new antifungal agents. The action spectrum of antifungal agents should be balanced, not too limited but also not too broad: effective against several species but not subject to early resistance. They should be stable and have limited off-target interactions and a known pharmacokinetic. The way of administration may be chosen preferring the patient compliance and considering hypothetical comorbidities. The choice between a complete eradication or just a control of the infection is crucial, especially in consideration of the problem of resistance and tolerance^20,21^.

Despite a lot of antifungal entities and new targets under investigation^17,20^, none of them has joined the market yet.

Among the novel therapeutic strategies, to treat fungal diseases, the employment of monoclonal antibodies (mAbs) appears as a great step forward. Monoclonal antibodies are promising therapeutic and diagnostic tools in different clinical contexts such as cancer, infective and autoimmune diseases^19^. Thanks to their high specificity for the determinant antigen, several mAbs were developed to treat fungal infections and reduce their bottlenecks but unfortunately none of them reached the clinical trials because of their murine nature (with the exception of Mycograb^22,23^).

Monoclonal antibody 2G8 is a successful murine mAb that showed in vitro activity against *Candida* spp. and *Aspergillus* spp. It showed a strong efficacy in vivo in a systemic mouse model of *Candida* infection and in a rat model of vaginal candidiasis^24^. The activity was verified also on *Cryptococcus neoformans*, confirming the capability to bind and inhibit the growth and capsule formation of this fungal species both in vitro and in vivo^25^. However, the murine source of this antibody precludes the possibility to use it in humans.

In this paper we reported the development and the characterization of a new humanized monoclonal antibody derived from mAb 2G8 and able to recognize β-1,3 glucans of pathogenic fungi such as *Candida* spp. These polysaccharides are vital components but hard to be reached because of the β-1,6-glucans, mannans and their glyco- and proteo-conjugates masking able to avoid the recognition by the host immune system. This new antibody has been obtained by the implementation of several different humanization procedures initially resulting in the selection of inactive candidates. By the combination of multiple approaches and after several attempts, we were finally able to select hmAb H5K1. It is stable and effective, at least in vitro. This antibody has also been tested in combination with several common antifungal drugs obtaining very interesting results.

## Results

### Humanization of the murine VH and VL from mAb 2G8

The study required different humanization approaches. The first protocol resorted to the CDR-grafting method^26^ and produced four sequences, with different degrees of backward mutations.

The humanization process based on the MG-score^27^ generated a trajectory that stopped at 42 mutations from the initial sequence in the second protocol (IMGT definition of the CDRs), and at 35 mutations in the third protocol (Kabat definition). Since the MG-score is inferred from a database of observed human variable-region sequences but does not include any explicit biophysical information on stability against aggregation, the most human-like sequence found is not guaranteed to be also the best hit for further development. For this reason, we used CamSol^28^ to study the expected solubility of the resulting trajectories and refine the choice of potential candidates to test in the laboratory, as explained in “Materials and methods” (Figs. S1, S2 in Supplementary Information reports the CamSol score).

Indeed, sequences starting from step 7 on both IMGT and Kabat trajectories are above the human-murine threshold score (see “Materials and methods”); on the other hand, the documentation of the CamSol Intrinsic tool states that scores larger than 1 denote highly soluble regions, while scores smaller than − 1 indicate poorly soluble ones. As a trade-off between the above considerations, we chose the VH sequence corresponding to sequence 19 and the VL of sequence 36 along the IMGT trajectory, and the VH of sequence 15 and VL of sequence 24 in the "Kabat" trajectory. Also, we considered the scFv corresponding to the VH and VL of sequence 19 in the IMGT case, and of sequence 15 in the Kabat case. Notice that in the latter sequences, the VHs are the same as those individually chosen before, but the VLs are different, so that we end up with only one choice of VH sequence and two choices of VL sequences for each trajectory. Table 1 reports the sequences obtained from both the humanization approaches.

**Table 1:** Identifiers of the heavy and light chains selected for further experimental inquiry, according to the protocol described in the text.

Heavy-chain sequence identifier	Position in trajectory (“I”:IMGT; “K”:Kabat; “C”: CDR-grafting)	Light-chain sequence identifier	Position in trajectory (“I”:IMGT; “K”:Kabat; “C”: CDR-grafting)
H1	C, 1	K1	C, 1
H3	C, 3	K3	C, 3
H5	I, 19	K5	I, 36
		K7	I, 19
H6	K,15	K6	K, 24
		K8	K,15

The identifier names are related to our internal naming scheme.

To identify the best binders of the humanized 2G8 antibody, the four different heavy chains (H_1_, H_3_, H_5_, H_6_) were combined with the seven different light chains (K_1_, K_3_, K_5_, K_6_, K_7_, K_8_) in a high-throughput microscale production system. The corresponding supernatants containing different antibodies were tested in ELISA assays for their binding to Laminarin.

**Figure 1:**
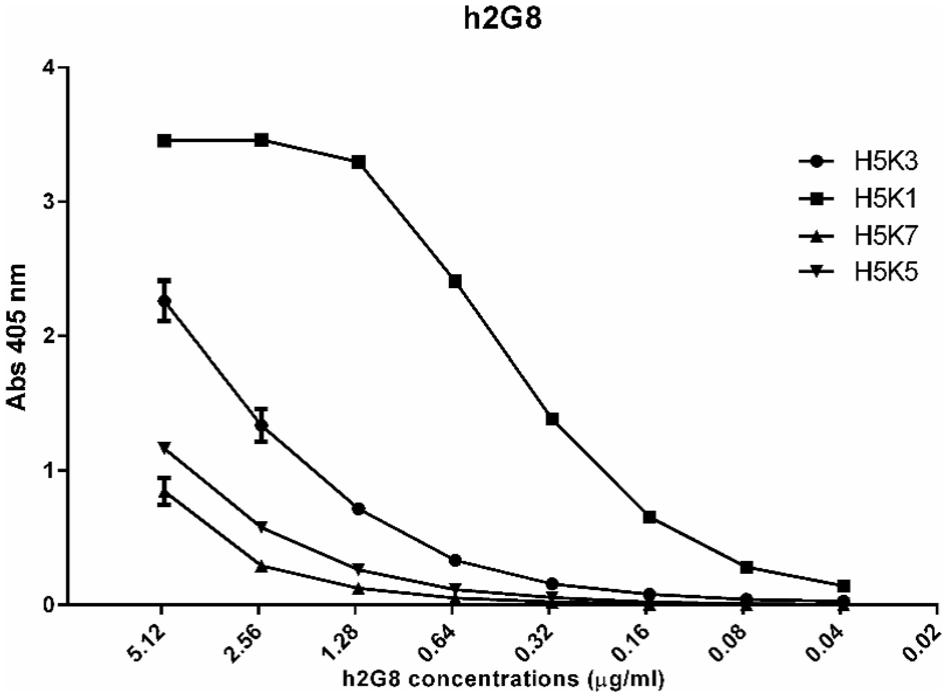
ELISA test performed to detect the activity of the four antibodies.

The four supernatants with the highest binding ability (H5 K1; H5 K3; H5 K5; H5 K7) (Fig. 1) were purified to confirm the previous results and, among these four antibodies (tested at 8 scalar dilutions (1:2) starting from 5 μg/ml), the combination heavy:light H5K1 was chosen because it was the best performer in the ELISA assay.

### Characterization of the humanized mAb H5K1

**Figure 2:**
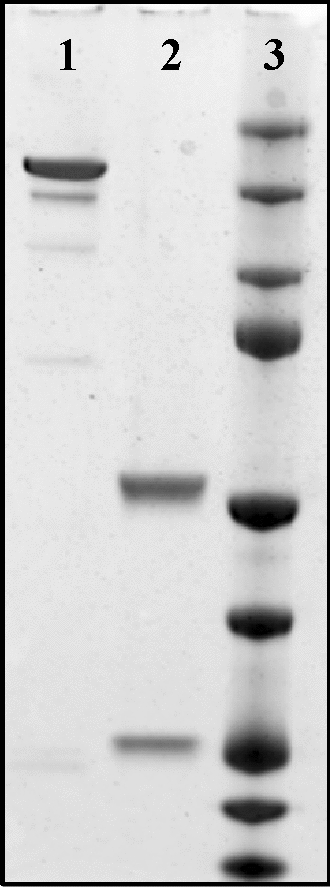
SDS-PAGE. 1: H5K1 not reduced, 2: H5K1 reduced, 3: Marker. Full-length blot is presented in Supplementary Fig. 2

**Figure 3:**
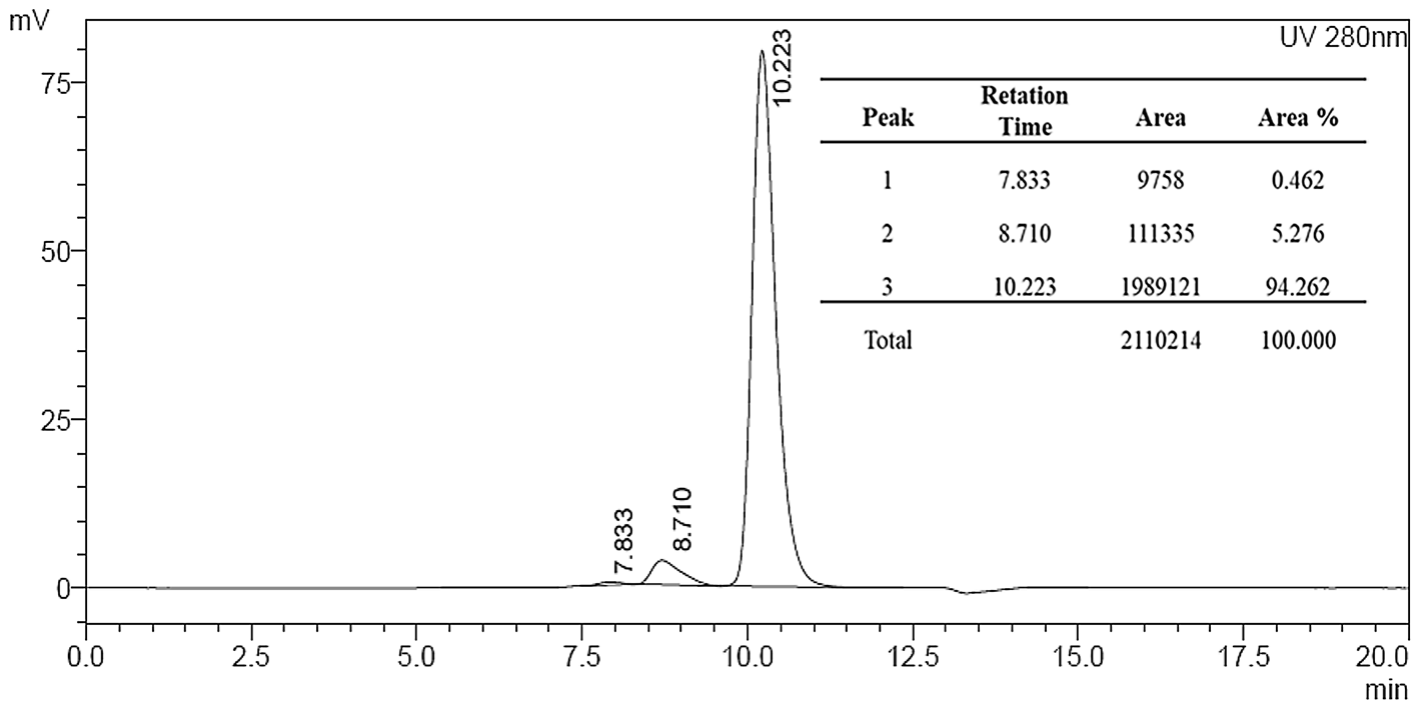
HPLC-SEC analysis of H5K1.

For the antibody characterization a medium scale production was executed using the EXPICHO Expression and it was purified by affinity chromatography. The structural integrity and the absence of aggregates were confirmed by SDS-PAGE (Fig. 2) and HPLC-SEC analysis (Fig. 3).

**Figure 4:**
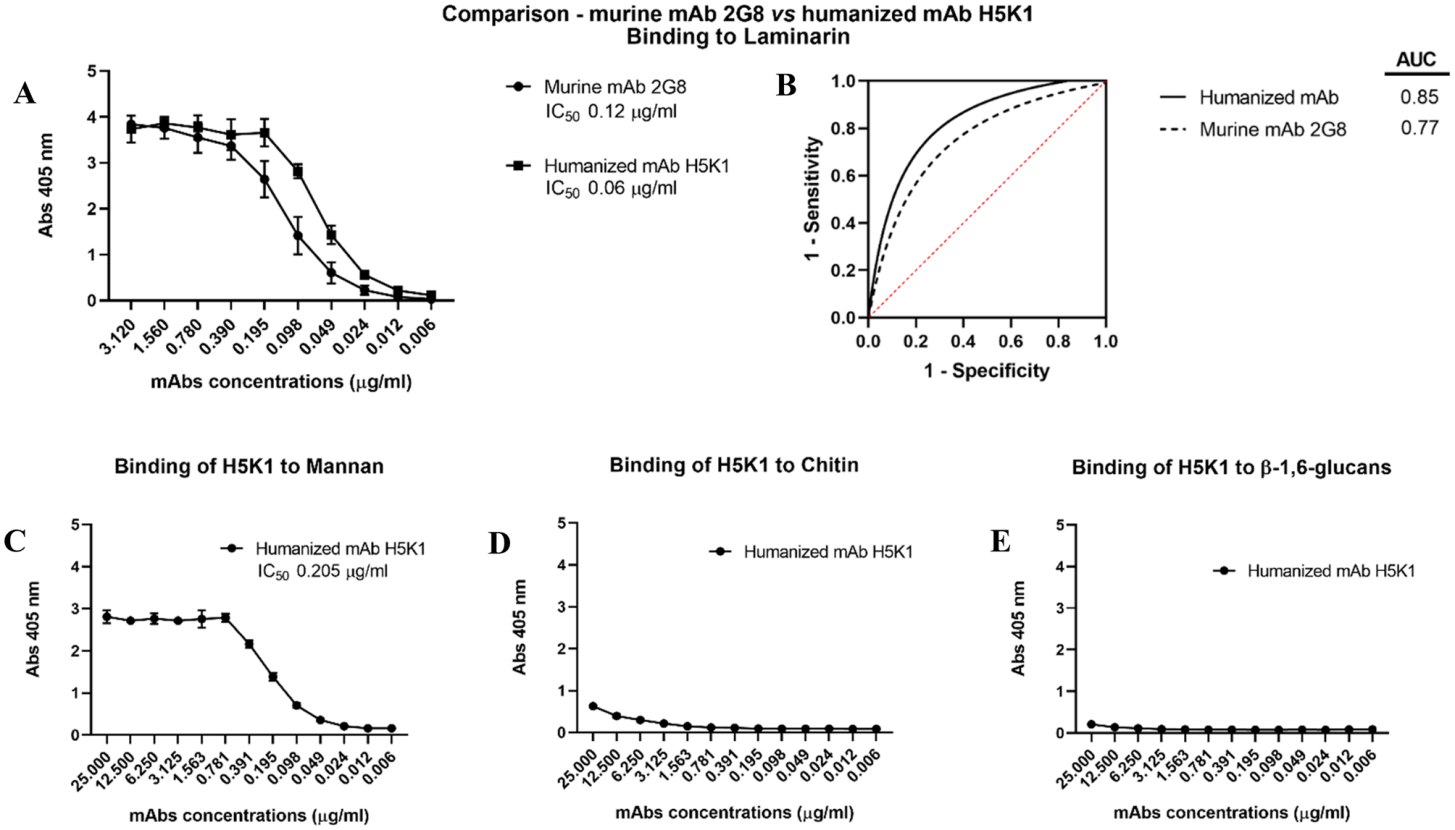
Binding to laminarin: comparison between the murine mAb 2G8 and the humanized H5K1 through ELISA assay (**A**) and the interpolated ROC curves of the ELISA assays’ mean (**B**). Binding to the other cell wall components: mannan (**C**), chitin (**D**) and β-1,6-glucans (**E**).

The purified mAb H5K1 was tested in ELISA first using laminarin as a target. Figure 4A shows the activity of the humanized H5K1 against laminarin compared to the murine 2G8. The IC_50_ values of the humanized and murine mAbs estimated from these data were respectively 0.06 and 0.120 µg/ml. This data, together with the predictive ability of the test performance of the interpolated ROC curves (Fig. 4B), their AUCs (0.85 for H5K1 and 0.77 for the murine 2G8) and the Kd evaluated with a competitive ELISA (3–5 × 10^–10^ M mean 4 with n = 4) support the superiority of the humanized antibody compared to the original murine one. In addition, in order to check the specificity, H5K1 was tested also with ELISA whose coating were respectively mannan, chitin and β-1,6-glucans extracted from *C. albicans* cells (Fig. 4C–E). It demonstrated to bind weakly chitin and β-1,6-glucans and only at higher concentrations while for what concerns mannan, H5K1 seems to bind but with an IC_50_ almost 3.5-fold higher than IC_50_ with laminarin (0.205 µg/ml). To further investigate the specific binding of H5K1 to β-1,3-glucans a competitive assay was performed through immunofluorescence. Fig. S3 in Supplementary Information reports a progressive decrease of H5K1 binding to *C. auris* cells with the increase of laminarin:H5K1 ratio.

### Flow cytometry and immunofluorescence

**Figure 5:**
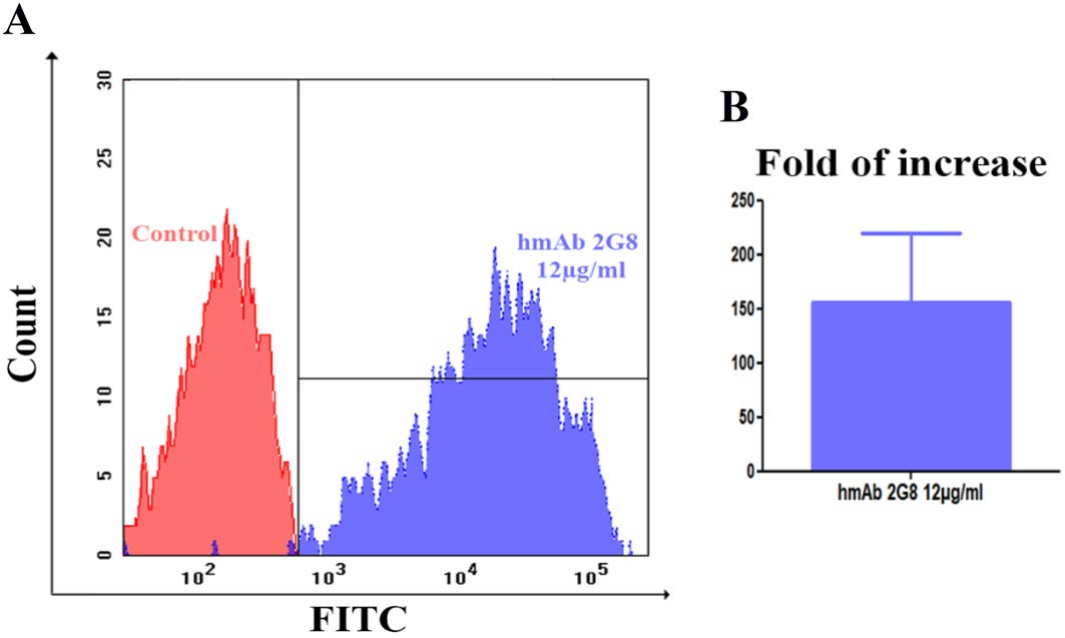
(**A**) Binding analysis by flow cytometry. Overlays of red histogram (from control sample) and blue histogram (from hmAb 2G8-labelled sample). (**B**) MFI fold of increase between the sample and the control.

Figure 5A enables us to quantify the brilliance/efficiency of the mAb, placing a marker defining the area of positivity for the molecule investigated (histogram referred to hmAb treatment). As highlighted, almost 100% of events express the antigen, however the net expression was precisely defined by Mean Fluorescence Intensities (MFI) values, taking into account the MFI values of hmAb 2G8 (blu histogram—MFI: 35932) by the ratio with the related MFI from FITC-conjugated secondary Ab (red histogram—MFI: 219) the fold of increase is 164, if compared to controls (Fig. 5B).

**Figure 6:**
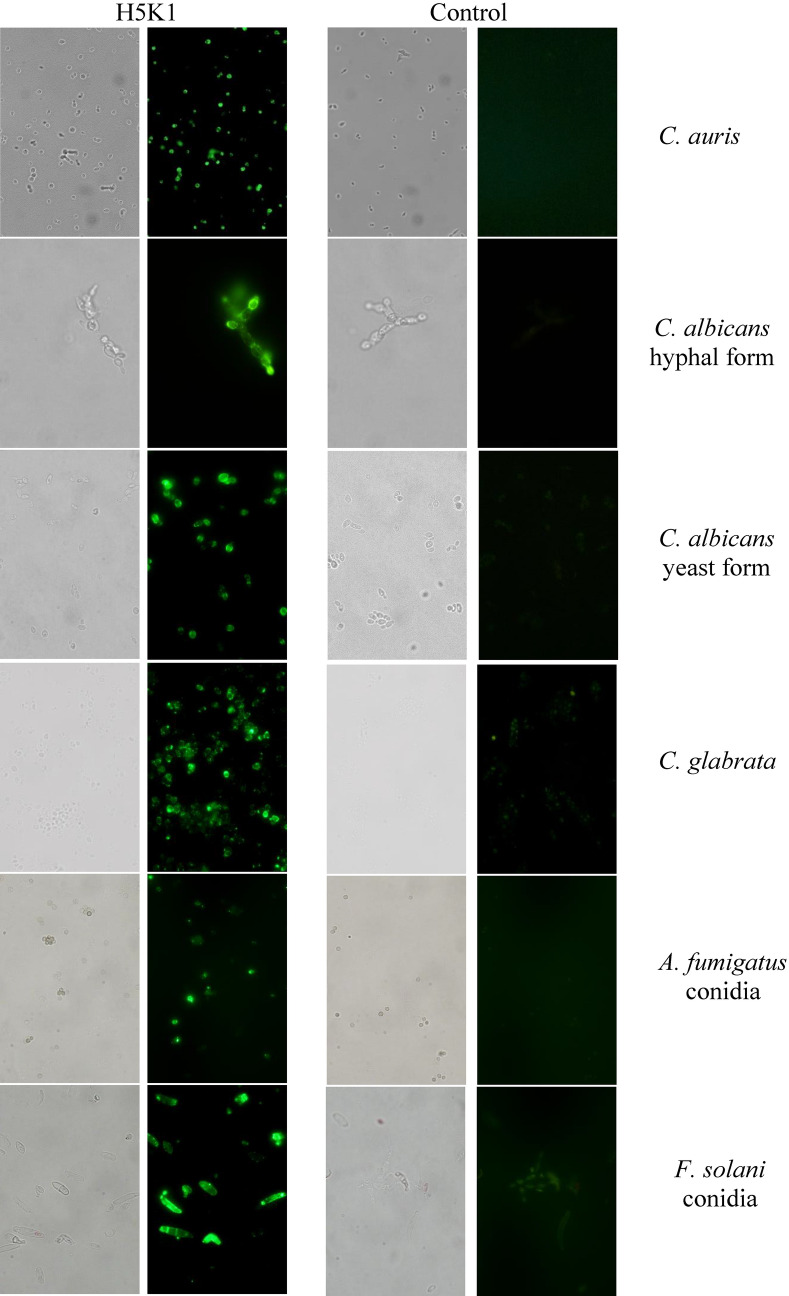
Binding analysis by means of immunofluorescence technique. Green fluorescence and bright-field images.

Several studies in murine models of candidemia using *C. albicans* showed increased representation of β-glucan and chitin in the cell wall during infection and drug treatment^29^. Although similar detailed studies are not yet available for *Candida auris*, the cell wall remains dynamic and can alter its structure depending on the environment and carbon source the fungi encounter, as well as in response to cell wall stress^30^. Flow cytometric (Fig. 5) and immunofluorescence analyses show the ability of the H5K1 to recognize and bind β-1,3-glucans on the surface of *C. auris* and highlight that hmAb is able to trace *C. auris* at a single cell level (without aggregation of fungal cells by the antibody presence; see Fig. S4 in Supplementary Information**)**, independently from the environment, following dynamic fluctuations of cell wall, as demonstrated by the histograms (that reveal different MFI values, but clearly define more than 97% of the sample). Moreover, apart from *C. auris,* H5K1 revealed to be able to bind also other *Candida* species such as *Candida albicans* both in hyphal and yeast form (see also S5 in Supplementary Information), and *Candida glabrata* (clinical isolate), but also *Aspergillus fumigatus* and *Fusarium solani* conidia (clinical isolates) (Fig. 6, Vid. 1A-B).

### Growth inhibition assay and adhesion assay

**Figure 7:**
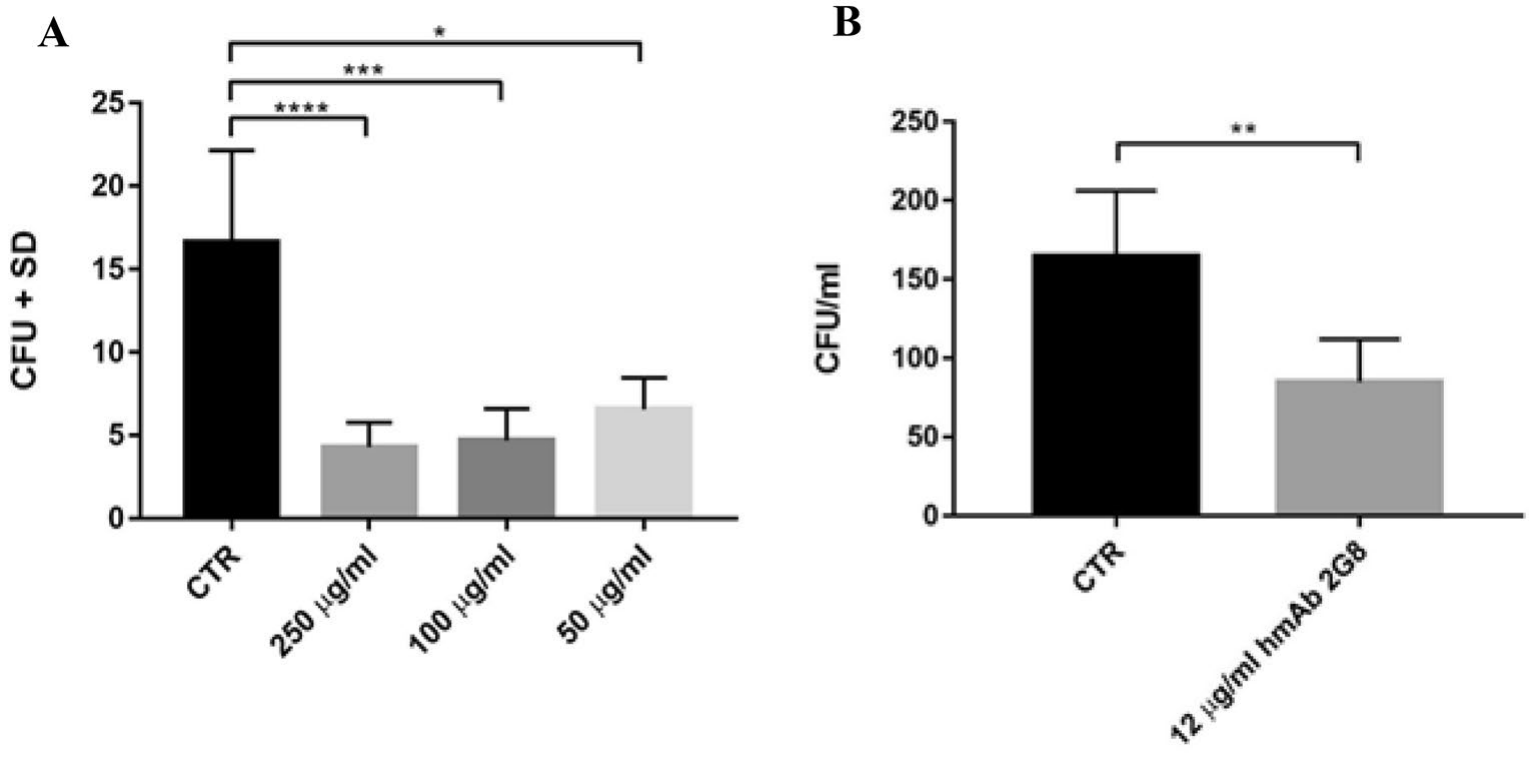
**A**) growth inhibition assay. **B**) adhesion assay. *p < 0.05, **p < 0.01, ***p < 0.001, ****p < 0.0001.

From the CFU counts, it appears that H5K1 provides a statistically significant effect on the inhibition of the *C. auris* cell growth at all the doses tested (Fig. 7A) ranging over 70% for 250 and 100 µg/ml and over 60% for 50 µg/ml and a statistically significant reduction in fungal adhesion to mammalian cells of 51.5% (Fig. 7B).

### Phagocytosis assay

**Figure 8:**
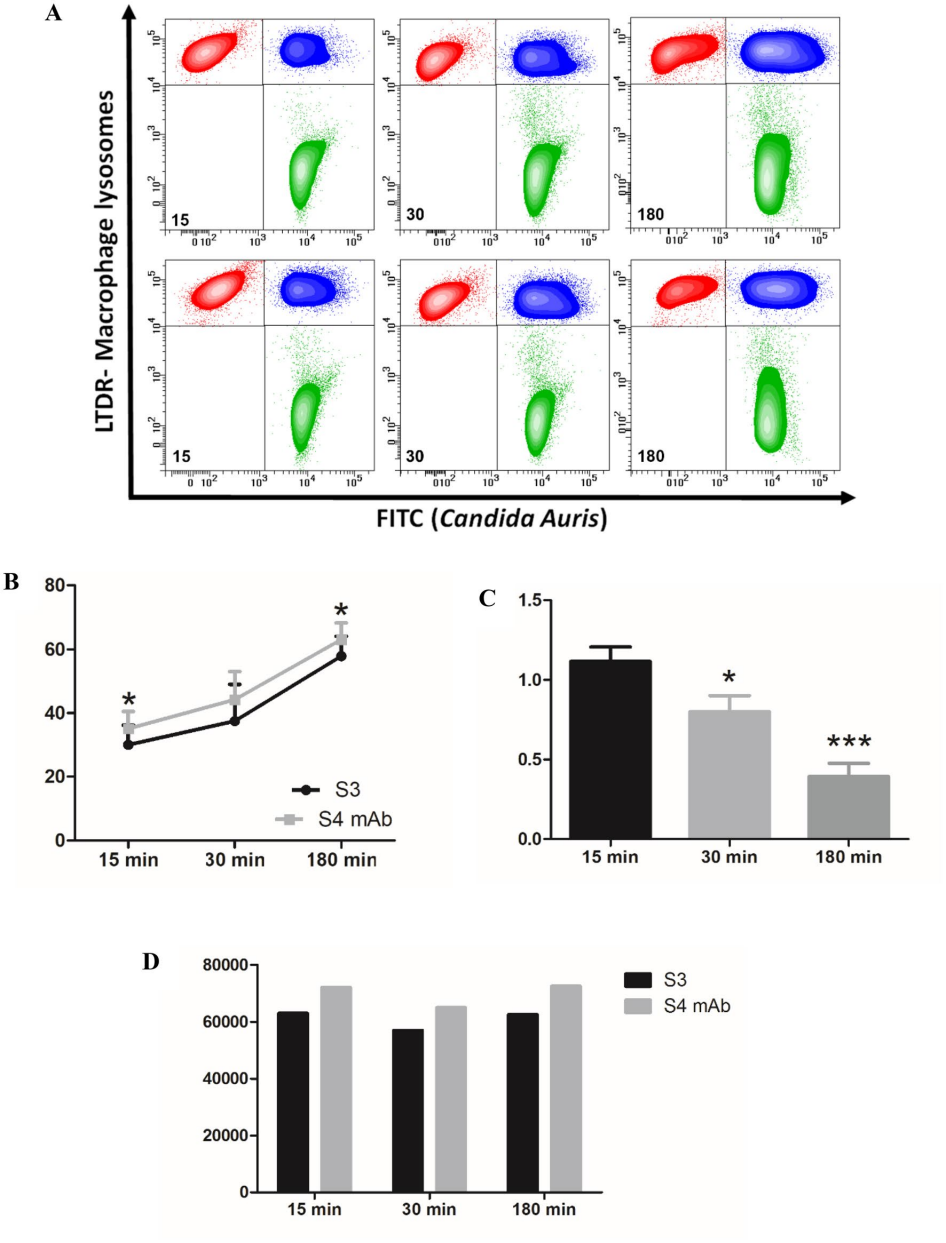
(**A**) FITC vs LTDR at 15, 30 and 180 min; in the upper plots *C. auris* cells were not pre-treated with the antibody while in the lower plots *C. auris* cells were pre-treated with H5K1. (**B**) Percentage of phagocyting macrophages. (**C**) Fold of decrease of residual *Candida auris* after treatment with the antibody. (**D**) Lysosome function.

Macrophages protect the body from damage and disease by targeting antibody-opsonized cells for phagocytosis. Contour plots FITC vs LTDR (Fig. 8A), illustrates the applied phagocytosis assay. In particular, LTDR labels Lysosomes of human macrophages, whereas FITC is the fluorescence used to trace *Candida auris*. The test was carried out after 15, 30 and 180 min from *Candida*/macrophages interaction, in a 1:1 ratio. Upper contour plots show the experiment related to *C. auris* not pre-treated with the antibody, whereas the bottom contour plots depict the experiment on *Candida* labelled by the unconjugated antibody. Briefly, red events represent not engulfing macrophages, blu events represent *Candida*-engulfing macrophages, and green events the residual free *Candida* in the samples. Results are statistically reported in scattergrams (Fig. 8B). As it is known, cooperative binding of antibody Fc domains to macrophage FcγR receptors triggers phagocytic cup formation in a process of adhesion and eventually internalization with phagosome closure. Scatter grams highlight a higher percentage of engulfing macrophages (Fig. 8B) for all the time points investigated, after labelling *Candida auris* with the antibody. In fact, opsonization of *C. auris* may lead to additional FcγRI binding, increased adherence and internalization, and enhanced phagocytosis. This cascade of events in antibody labelled samples is confirmed also by residual *Candida auris* which undergoes a reduction at each time point, with the best result of more than half reduction after 180 min (Fig. 8C). This highlights that the H5K1 immunomodulatory effects are not restricted to the percentage of actively phagocyting macrophages. Finally, as last consideration, in hmAb-pre-treated samples, macrophages seem more efficient, as demonstrated also by the functionality of their lysosomes (Fig. 8D) in fact, it is known, that induced damages to macrophage lysosome crucially contribute to fungal virulence^31^.

### Minimal inhibitory concentration (MIC) assays

**Figure 9:**
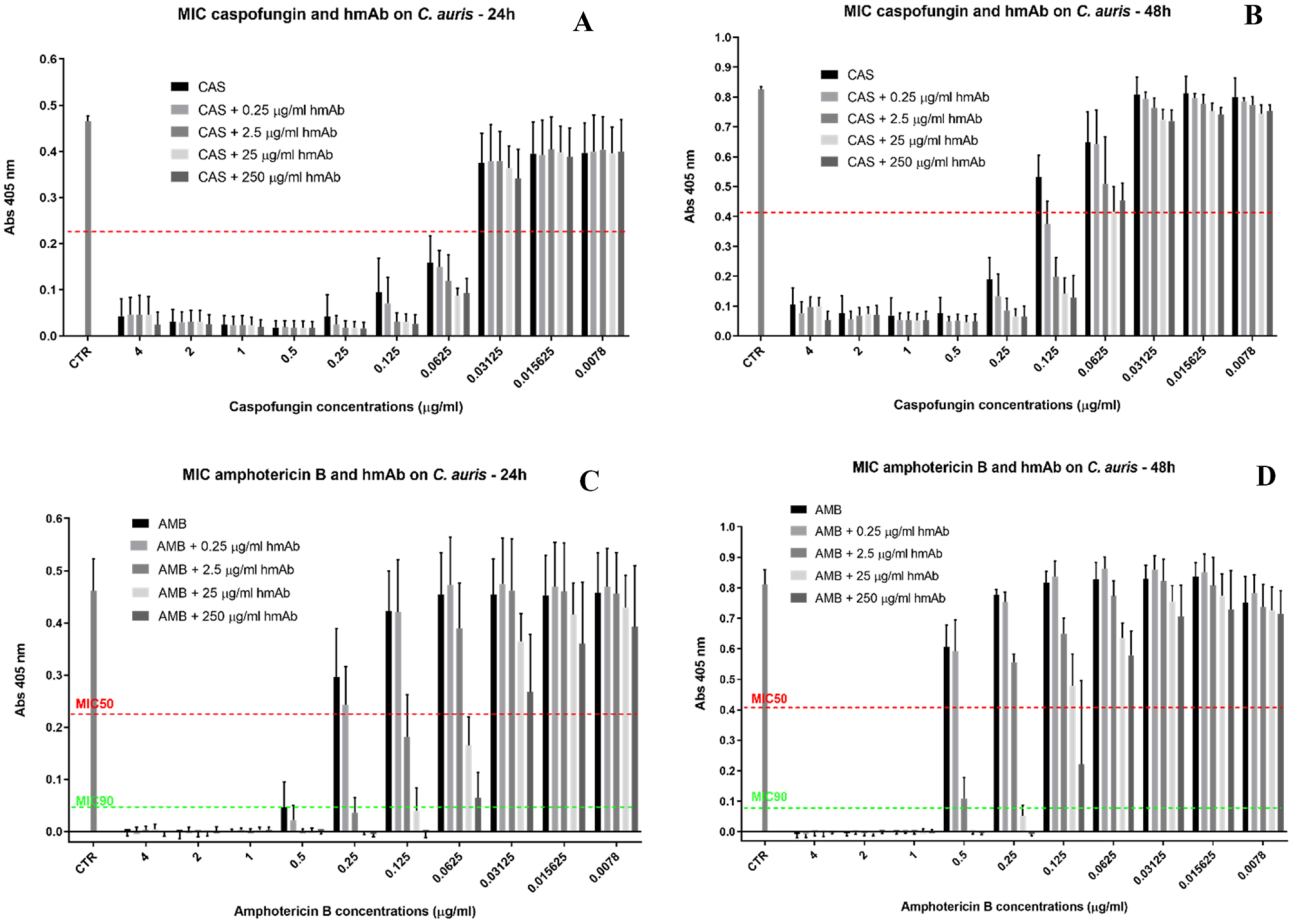
Figure 8A,B, MIC assays of caspofungin alone and in combination with different concentrations of humanized H5K1 at 24 and 48 h. Figure 8C,D, MIC assays of amphotericin B in combination with different concentrations of humanized H5K1 at 24 and 48 h.

Figure 9 shows the results of MIC assays obtained by testing antifungals caspofungin (CAS) and amphotericin B (AMB) alone and in combination with hmAb H5K1 against *C. auris*. With the term MIC50 we refer to the lowest concentration that inhibits the 50% of the growth compared to drug-free control and with MIC90, the lowest concentration that inhibits the 90% of the growth compared to drug-free control. After 24 h (Fig. 9A) the MIC50 (red line) breakpoints of CAS alone and in combination with H5K1 are found at 0.0625 µg/ml with little dependence on H5K1 concentration. On the contrary at 48 h (Fig. 9B), the action of H5K1 is more pronounced, and MIC50 is established at 0.25 µg/ml for CAS alone, while at 0.125 µg/ml when in combination with the antibody. This corresponds to a reduction of 1 dilution of CAS concentration (from 0.25 to 0.125 µg/ml). CAS and the humanized mAb are assumed as synergic by definition^32,33^. For AMB, at 24 h (Fig. 9C), the drug alone has a MIC50 (red line) and MIC90 (green line) at 0.5 µg/ml whereas, with 2.5 µg/ml of H5K1, MIC50 and MIC90 are respectively at 0.125 µg/ml and 0.25 µg/ml. Notably with 25 and 250 µg/ml of H5K1, MIC50 shifts at 0.0625 and MIC90 at 0.125 µg/ml. At 48 h (Fig. 9D), AMB alone has MIC50 and MIC90 at 1 µg/ml, while with 2.5 µg/ml of hmAb, MIC50 decreases to 0.5 µg/ml. The combination with 25 µg/ml of hmAb fixes MIC50 and MIC90 together at 0.25 µg/ml while with 250 µg/ml of the humanized mAb, MIC90 is at 0.25 µg/ml and MIC50 at 0.125 µg/ml. In Tables 2, 3, 4 and 5 are reported the percentage of inhibition at 24 and 48 h.

**Table 2:** Percentage of growth inhibition at 24 h of CAS and CAS in combination with H5K1.

Drug µg/ml	CAS	CAS in combination with:			
		0.25 µg/ml H5K1	2.5 µg/ml H5K1	25 µg/ml H5K1	250 µg/ml H5K1
4	90.3	89.7	89.6	89.6	94.5
2	93.1	93.3	93.1	93.0	94.2
1	94.5	94.7	94.6	94.8	95.7
0.5	95.8	95.7	95.9	95.8	96.1
0.25	90.4	94.4	96.0	95.8	96.2
0.125	79.0	94.0	93.1	93.1	94.0
0.0625	68.1⁎	69.1⁎	77.1⁎	80.4⁎	79.3⁎
0.03125	16.5	15.6	15.4	18.9	23.8
0.0156	11.9	12.6	9.8	11.3	13.3
0.0078	11.5	11.0	9.9	11.7	10.8

The MIC50 breakpoints are marked with *.

**Table 3:** Percentage of growth inhibition at 48 h of CAS and CAS in combination with H5K1.

Drug µg/ml	CAS	CAS in combination with:			
		0.25 µg/ml H5K1	2.5 µg/ml H5K1	25 µg/ml H5K1	250 µg/ml H5K1
4	89.6	90.8	88.3	88	93.4
2	93	93.2	91.7	91.1	91.4
1	93.9	93.5	93.5	93.7	93.6
0.5	93	94.1	93.7	94.4	94.1
0.25	79.1⁎	86.7	89.6	92	92
0.125	37.6	54.6⁎	76⁎	82.8⁎	84.5⁎
0.0625	23.7	22.1	38.3	49.6	45.1
0.03125	4.4	3.8	7.5	12.3	12.8
0.0156	3.8	3.6	5.8	8.9	10.3
0.0078	5.3	4.9	6.3	10	8.8

The MIC50 breakpoints are marked with *.

**Table 4:** Percentage of growth inhibition at 24 h of AMB and AMB in combination with H5K1.

Drug µg/ml	AMB	AMB in combination with:			
		0.25 µg/ml H5K1	2.5 µg/ml H5K1	25 µg/ml H5K1	250 µg/ml H5K1
4	98.8	99.8	98	97.8	98.7
2	99.8	99.4	99.8	99.8	99.1
1	100	99.8	100	100	99.8
0.5	90.7⁎,^**†**^	95.2⁎,^**†**^	100	100	100
0.25	35.1	46.6	91.7^**†**^	99.6	100
0.125	7.2	8.3	60.4⁎	91.3^**†**^	100^**†**^
0.0625	0	0	13.5	62.6⁎	84.8⁎
0.03125	1.1	0	0	20.7	41.8
0.0156	1.8	0	0	9.6	21.6
0.0078	0.5	0	0	6.5	15

The MIC50 breakpoints are marked with *. The MIC90 breakpoint are marked with ^†^.

**Table 5:** Percentage of growth inhibition at 48 h of AMB and AMB in combination with H5K1.

Drug µg/ml	AMB	AMB in combination with:			
		0.25 µg/ml H5K1	2.5 µg/ml H5K1	25 µg/ml H5K1	250 µg/ml H5K1
4	100	100	100	100	100
2	100	100	100	100	100
1	100⁎,^**†**^	100*,^**†**^	100^**†**^	100	100
0.5	25.5	27.2	86.7*	100	100
0.25	4.4	7.3	31.6	93.5*,^**†**^	100^**†**^
0.125	0	0	20	41.1	72.8*
0.0625	0	0	4.9	21.7	28.7
0.03125	0	0	0	7.2	13
0.0156	0	0	0.6	4.7	10.4
0.0078	7.6	3.7	9.1	10.6	12

The MIC50 breakpoints are marked with *. The MIC90 breakpoint are marked with ^†^.

### Time-kill curve assay

**Figure 10:**
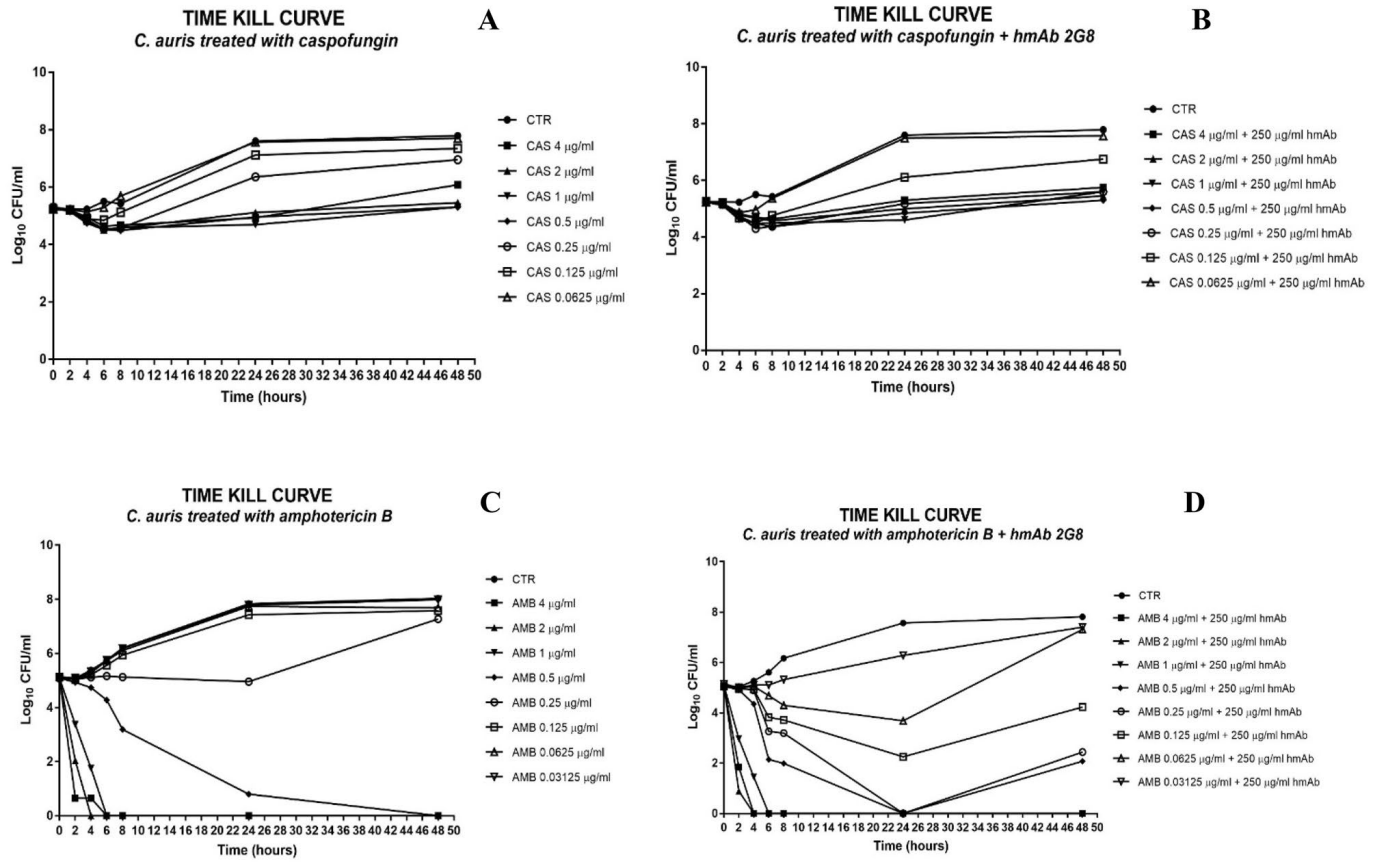
Time-kill curve of caspofungin (**A**,**B**) and amphotericin B (**C**,**D**) alone and in combination with H5K1.

A time-kill curve based on the data from MIC assays was elaborated to evaluate how the H5K1 combined with CAS and AMB could improve their basal activity on *C*. *auris* (Fig. 10). At 24 h and 48 h, when at 0.25 and 0.125 µg/ml CAS efficiency starts decreasing (Fig. 10A), the presence of hmAb ameliorated the drug efficiency. In particular, the combination of 0.25 µg/ml CAS + 250 µg/ml H5K1 maintained a constant fungistatic trend all over time, while the combination with 0.125 µg/ml CAS resulted in a recovery slower than with CAS alone (Fig. 10B). The combination of CAS 0.25 µg/ml and H5K1 ensures more than 1log difference both at 24 and 48 h and the combination with 0.125 µg/ml shows more than 1log difference at 24 h and more than half log at 48 h as reported in Table 6. Coming to AMB, we observe that even if it is a fungicidal, its efficiency alone starts decreasing at doses lower than 0.5 µg/ml and it is visible only after 24 h (Fig. 10C). The presence of H5K1 prompts for an earlier efficacy already at 6 h as reported in Table 7. Moreover, the combination shows a better effect with almost every concentration of AMB than the drug alone, in terms of cells revival with a reduction still ≥ 3 log at 24 h with AMB 0.25 µg/ml (Fig. 10D). According to the standardized method^34^, the synergic effect between two agents is revealed with a difference ≥ 2 log in their respective growth decreases after 24 h. The strong synergy between AMB and H5K1 is evident from data shown in Table 8.

**Table 6:** **∆**log of the respective samples with the starting inoculum as evaluation of hmAb contribution in combination treatment**.**

	24 h	48 h
CAS 0.25 µg/ml	− 1.11	− 1.71
CAS 0.25 µg/ml + 250 µg/ml H5K1	− 0.02	− 0.46
CAS 0.125 µg/ml	− 1.88	− 2.10
CAS 0.125 µg/ml + 250 µg/ml H5K1	− 0.87	− 1.51

**Table 7:** ∆log of the samples with the starting inoculum.

	0 h	2 h	4 h	6 h	8 h	24 h	48 h
AMB 4 µg/ml	0.06	** ≥ 3Log**	** ≥ 3Log**	** ≥ 3Log**	** ≥ 3Log**	** ≥ 3Log**	** ≥ 3Log**
AMB 4 µg + 250 µg/ml H5K1	0.08	** ≥ 3Log**	** ≥ 3Log**	** ≥ 3Log**	** ≥ 3Log**	** ≥ 3Log**	** ≥ 3Log**
AMB 2 µg/ml	0.05	** ≥ 3Log**	** ≥ 3Log**	** ≥ 3Log**	** ≥ 3Log**	** ≥ 3Log**	** ≥ 3Log**
AMB 2 µg + 250 µg/ml H5K1	0.10	** ≥ 3Log**	** ≥ 3Log**	** ≥ 3Log**	** ≥ 3Log**	** ≥ 3Log**	** ≥ 3Log**
AMB 1 µg/ml	0.05	*1.77*	** ≥ 3Log**	** ≥ 3Log**	** ≥ 3Log**	** ≥ 3Log**	** ≥ 3Log**
AMB 1 µg + 250 µg/ml H5K1	0.05	*2.16*	** ≥ 3Log**	** ≥ 3Log**	** ≥ 3Log**	** ≥ 3Log**	** ≥ 3Log**
AMB 0.5 µg/ml	0.05	*0.21*	*0.41*	*0.86*	*1.95*	** ≥ 3Log**	** ≥ 3Log**
AMB 0.5 µg + 250 µg/ml H5K1	0.09	*0.22*	*0.78*	** ≥ 3Log**	** ≥ 3Log**	** ≥ 3Log**	** ≥ 3Log**
AMB 0.25 µg/ml	0.07	*0.10*	*0.02*	*− 0.02*	*0.02*	*0.19*	*− 2.13*
AMB 0.25 µg + 250 µg/ml H5K1	0.11	*0.13*	*0.25*	*1.88*	*1.95*	** ≥ 3Log**	*2.66*
AMB 0.125 µg/ml	0.01	*0.11*	*− 0.07*	*− 0.42*	*− 0.80*	*− 2.28*	*− 2.44*
AMB 0.125 µg + 250 µg/ml H5K1	0.09	*0.20*	*0.21*	*1.31*	*1.42*	*2.88*	*0.91*
AMB 0.0625 µg/ml	0	*0.07*	*− 0.14*	*− 0.59*	*− 0.97*	*− 2.59*	*− 2.57*
AMB 0.0625 µg + 250 µg/ml H5K1	0.09	*0.15*	*0.09*	*0.46*	*0.84*	*1.45*	*− 2.17*
AMB 0.03125 µg/ml	0.01	*0.05*	*− 0.19*	*− 0.62*	*− 1.04*	*− 2.65*	*− 2.85*
AMB 0.03125 µg + 250 µg/ml H5K1	0.07	*0.13*	*0.01*	*0.03*	*− 0.18*	*− 1.14*	*− 2.26*

The fungistatic effect is in italic and the fungicidal in bold.

**Table 8:** **∆**log between amphotericin B effect and the corresponding combination with H5K1.

AMB concentrations	∆log: AMB − AMB + 250 µg/ml H5K1	
	24 h	48 h
0.25 µg/ml	4.95	4.79
0.125 µg/ml	5.16	3.35
0.0625 µg/ml	4.04	0.4

## Discussion

Invasive fungal infections affect mainly patients undergoing transplantation, surgery, neoplastic disease, immunocompromised subjects and premature infants and cause over 1.5 million deaths every year. Their treatment is challenging, with drugs’ toxicity and acquired and innate fungal resistance representing the major hurdles to overcome^20^.

Monoclonal antibodies are powerful tools currently used as diagnostic and therapeutic agents in different clinical contexts. With over 60 monoclonal antibodies in late-stage clinical studies and an estimated global market of $125 billion, these biological drugs are the main candidates of the future medicine as the current circumstance of Covid-19 pandemic has taught us^35–39^. The humanized mAb H5K1 presented in this work and derived from the murine mAb 2G8^24,25^, is able to recognize and bind selectively β-1,3-glucans in the fungal wall. Several key points emerging from our findings can be highlighted: (1) first of all, from the methodological point of view, this study represents the first experimental test of the humanization protocol proposed in^27^. Our findings suggest that the approach is not perfect and there is margin for further improvement: the overall best sequence scores poorly in CamSol solubility score (so we suspect that it is aggregation-prone, even though we have not tested it experimentally); moreover, the sequence H5K1, that we have finally chosen for further analysis, being the best performer in ELISA assay, is a combination of a heavy chain obtained by MG-optimization, and a light chain obtained by CDR-grafting. On the other hand, all the four best-performing sequencies in the ELISA assay have the heavy chain found by MG-optimization, and the third and fourth of them also have the light chain found by this protocol; therefore, the MG approach, especially when combined with Camsol for further refinement, appears as an interesting tool to identify candidate hits for humanization.

(2) Second, the humanization process has not changed the capability of the mAb to bind the antigen as shown in the ELISA assay. Furthermore, experimental outputs revealed that H5K1 performs better than the original murine 2G8. The binding activity is confirmed also in in vitro assays on *C. auris, C. albicans, C. glabrata* cells and on *Aspergillus fumigatus and Fusarium solani* conidia, as demonstrated by microscopy and flow cytometry analyses. Intrigued by those promising results, we assessed whether the binding of hmAb could affect cells growth and adhesion, as it is the case for the murine mAb^40^. As reported in “Results” section, in growth inhibition and adhesion assays mAb H5K1 displayed an activity which confirms the positive effects already obtained with the murine 2G8;

(3) Third, the opsonization activity of H5K1 to *C. auris* cells determines an improvement of the engulfment by macrophages which result more efficient as shown by their active lysosomes compared to the controls and from the residual *C. auris* that decreases significantly with the passing of time.

(4) Finally, we found that the co-administration of our antibody with some commercially available antifungal drugs could provide an important benefit. FLC, CAS and AMB were chosen as the best representatives of their classes and tested with and without mAb H5K1 on *C. auris*. While with FLC we obtained just a little difference upon adding the antibody (data not shown), in combination with CAS and AMB, hmAb played an essential role in terms of concentration required to reach the desired effect and time of action. Well known are the side effects and the toxicity of AMB especially at high concentrations^41^, hence the possibility to reduce the therapeutic doses is a hopeful perspective, especially for patients’ compliance and treatment adherence. CAS is more tolerated and safer, but some studies highlighted negative drug-drug interactions and, even worse, CAS (as well as other antifungals) are blamed to act as selectors of resistant species when used at too low concentrations^42^.

In the MIC assay the combination mAb H5K1-CAS, synergic by definition, brings to a 1:2 dilution concentration shift at 48 h, which was also found in the time kill curve, where the hmAb makes the fungistatic effect still effective and preserved at very low concentrations all over time. When combined with amphotericin B, the hmAb had a more incisive impact both at 24 and 48 h, but time kill curve was essential to define the presence of a synergism. The combination performs always better than the drug alone and the fungicidal effect is visible earlier and at concentrations at which amphotericin B alone has already lost it. We believe there is an important contribution of our hmAb even at earlier time points and at higher drug concentrations, although we cannot detect this effect, because of the heavy fungistatic and fungicidal effects of drugs alone against a non-resistant strain.

Notably, the combinations could have a fundamental role in the treatment of resistant species where the drugs alone have little or no effects even at high doses. Further studies are needed to test this hypothesis, as well as to check the action on other fungal strains and in vivo.

In view of the results obtained, we are optimistic that our new humanized mAb H5K1 could be a substantial player in the fight against fungal infections since it has all the qualities to reach clinical trials both alone and in combination with other drugs.

## Materials and methods

### Cell lines

in our studies we used *C. auris* (strain DMS 21092) as fungal model, *Candida albicans* (ATCC 10231), clinical isolates of *Candida glabrata*, *Aspergillus fumigatus* and *Fusarium solani,* cervical carcinoma HeLa cells (ATCC) and CHO cells (ExpiCHO-S Cells, Thermo Fisher Scientific).

### Humanization process

An analysis of murine 2G8 VH and VL against IgBlastTool^43^ murine databases (IMGT mouse V genes, IMGT mouse D genes, IMGT mouse J genes) brought to the substitution of the non-homologous amino acids to decrease the value of the *instability index* calculated with ExPasy^44^. The modified murine VH and VL underwent CDR-grafting following two methodologies: the first analysed the whole VH and VL sequences, the second just the Framework Regions (FRW). The latter method produced the VH and VL sequences with the lowest instability index and for this reason they were compared with the most similar ones in PDB, and the non-homologous amino acids were substituted. Some amino acids were back mutated to the murine ones.

**Figure 11:**
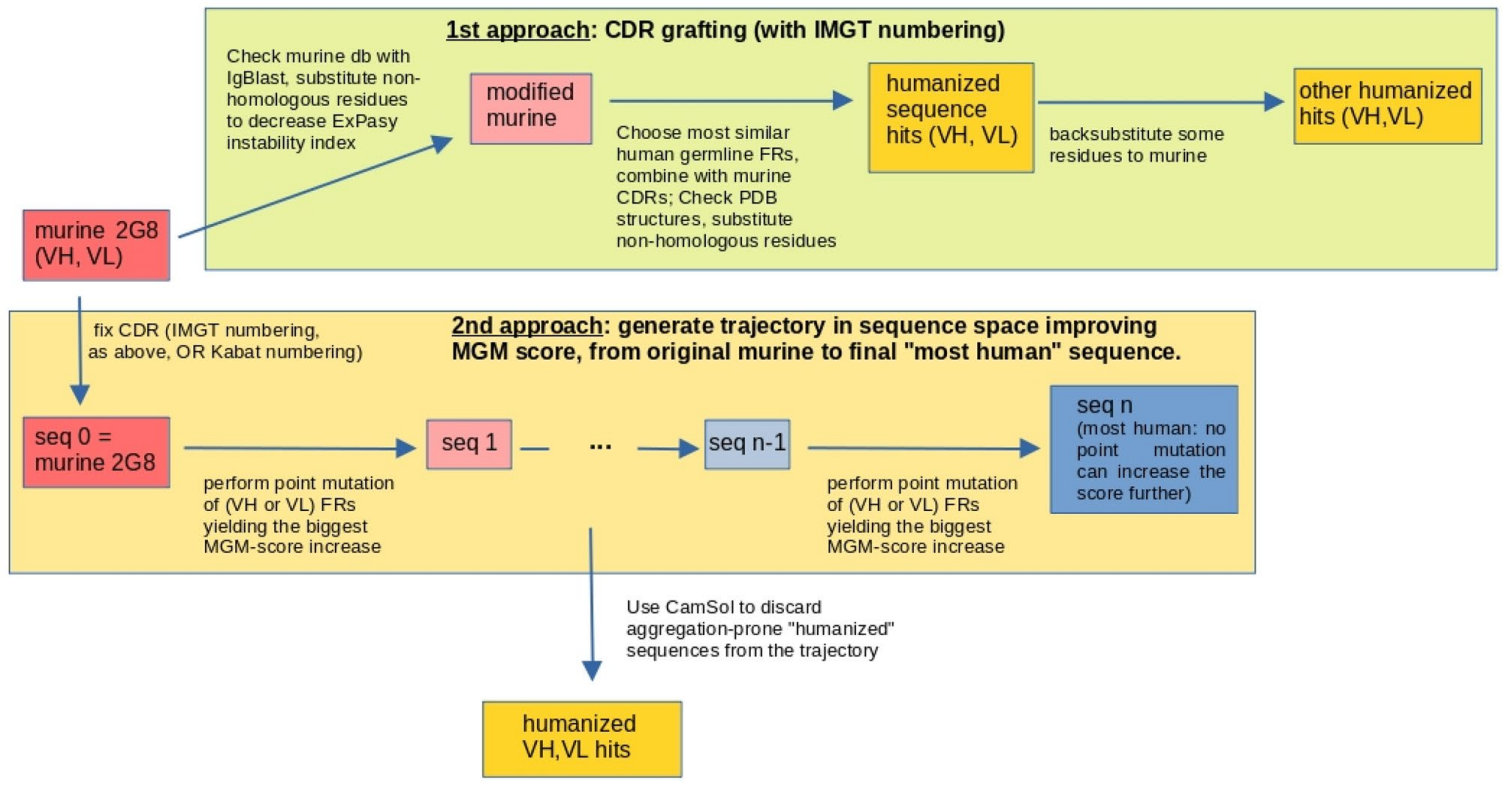
Flowchart of the humanization process: humanized sequences (in gold) are obtained according to either CDR grafting (above) or iterated MGM-score-based mutations (below); the latter approach yields a trajectory of increasingly human sequences from the original, murine one (red) to the final sequence, that cannot be further improved under point mutations (blue). CamSol Intrinsic server is used to select, among the sequence on this trajectory, the humanized hits as the ones that are sufficiently human and sufficiently soluble.

The other two protocols use the MG-score and the humanization approach introduced in^27^, resorting to statistical modelling of the variable regions of human antibody sequences: VH and VL regions were aligned according to the AHo scheme and juxtaposed, to yield a unique VH-VL sequence of length 298 amino acids (including gaps); then, the two protocols just differ in the choice of the CDR regions to be kept fixed: in one case, they coincided with residues chosen for the CDR-grafting protocol above, while in the second case, CDR residues were those corresponding to the Kabat numbering scheme. In Fig. 11 we show a schematic representation of both the CDR-grafting and MGM humanization process. This approach, that includes residue-residue correlations both within and between heavy and light chains, strongly depends on which residues are kept fixed, as belonging to CDR regions: starting from the murine sequence, the method implies mutating one residue at a time, choosing the mutation (at any site and to any amino-acid) that yields the greatest increase in the MG-score. This method does not guarantee to reach the highest score (i.e. the most humanlike sequence with the given CDRs), but it finds the local maximum closest to the initial murine sequence, with the smallest number of mutations (i.e. the shortest path to it). The trajectories in sequence space thus produced contain several sequences that are above the human-murine threshold^27^ and thus constitute potential hits. These sequences were further analysed with CamSol Intrinsic Server^28,45^, to check explicitly their tendency towards aggregation. Since CamSol uses local sequence information for prediction, the latter could be affected by the presence of the artificial contiguity between the C-term of the VH chain and the N-term of the VL chain in the alignment used in the design step above: for this reason, we analysed separately the VH and VL chains with CamSol, and also the scFv constructs, with a common linker between the two chains. This allows to pick, along the Kabat trajectory, separately the VH and VL sequences that have the least tendency towards aggregation, among those that score above the murine/human threshold, and also, the scFv that best copes with the requirements of having a good MG-score and a good solubility; the same was done for the other protocol, with the IMGT trajectory.

Having observed^27^ that the correlations between VH and VL are quite smaller than correlations within VH and VL regions, we consider, for further experimental testing, the VH-VL combinations corresponding to the scFv sequences, and also the constructs obtained by combining in all possible ways the 2 VH and VL sequences thus obtained, together with those obtained with the CDR-grafting protocol. In the end, excluding repetitions, a total of 28 combinations of VH and VL were used to develop new full-length antibodies.

### Antibody production

Recombinant antibodies were constructed in vitro using recombinant DNA technology. Antibody heavy and light variable fragments were synthesized based on sequences information obtained after humanization. They were then cloned into vectors for the expression in eukaryotic system and containing the human IgG1 heavy chain or the kappa light chain constant regions to create full length heavy and light chain constructs. Full length IgG1 heavy and kappa light chain genes were co-transfected in a high-throughput microscale system. 3 ml of CHO-S cells culture was transfected using the EXPICHO Expression system (Thermo Scientific™) according to the information supplied by the manufacturer. After 1 week, supernatants were collected for further analysis. The antibody production on a medium scale was carried out with the same expression system.

### Antibody purification

Supernatants containing antibodies were centrifuged, filtered 0.22 μm to remove cells and debris and batch purified by affinity chromatography using TOYOPEARL AF-rProtein A HC-650F resin (Tosoh Bioscience LLC).

Antibodies were eluted with Buffer Citrate 0.1 M pH 3, neutralized with an alkaline solution and dialyzed in PBS1X with slide-A-Lyzer (Thermo Scientific™). Purity of the antibody was checked by SDS-PAGE analysis that was performed under reducing/non-reducing conditions according to the standard method. Endotoxin levels were evaluated by Pierce™ LAL Chromogenic Endotoxin Quantitation Kit (Thermo Scientific™) and the presence of aggregates analysed through HPLC-SEC analysis.

### Mannan, chitin and β-1,6-glucans extraction

From an overnight inoculum of *C. albicans*, the cells were extracted with 3% NaOH for 1 h at 80 °C. The suspension was centrifuged; the alkali-extracted supernatant was used for the precipitation of mannan through Fehling’s reaction while the pellet was digested overnight with Kitalase lytic enzyme (FUJIFILM Wako Pure Chemical Corporation) containing β-1,3-glucanase but not chitinase or β-1,6-glucanase. The digested product was centrifuged: the supernatant composed mainly by the remaining β-1,6-glucans was dialysed against 10 mM Tris–HCl (pH 7.5) to remove digested β-1,3-glucanas while the precipitate representing chitin was solubilized. Phenol–sulfuric acid method was used for the polysaccharides quantification^46^.

### Competitive binding in immunofluorescence

12 µg/ml of H5K1 was added to different concentrations of laminarin in PBS 3% BSA in order to reach the following laminarin:H5K1 ratios: 40:1, 16:1, 4:1, 1:1 and 0:1. The solutions were left react at 37 °C for one hour then were put in contact with 3.0 × 10^6^ *C. auris* cells coming from an overnight inoculum. After 1 h the cells were centrifuged, washed, and marked for 1 h with anti-human IgG FITC antibody (Abcam, ab97224) 1:150 in PBS + 3% BSA. The cells were washed before and after fixing with paraformaldehyde (Carlo Erba) 4% for 1 h at 4 °C and finally they were resuspended in 100 µl of PBS. The samples were analysed through immunofluorescence microscope.

### ELISA assay

96-well plates were coated with 50 μg/ml laminarin (Sigma-Aldrich, L9634) or extracted mannan, chitin and β-1,6-glucans in 0.05 M carbonate buffer pH 9.6 overnight at 4 °C. Nonspecific interactions were blocked with 100 µl/well blocking solution, 3% (w/v) BSA in PBS-Tween 20 (8 g/l NaCl, 0.2 g/l KH_2_PO_4_, 2.9 g/l Na_2_HPO_4_·12H_2_O, 0.2 g/l KCl, 0.05% (v/v) Tween 20, pH 7.4) at 37 °C for 1 h. The plates were then incubated with decreasing concentrations of humanized mAb 2G8 (from 3.12 to 0.006 μg/ml, each concentration was tested in triplicate) in blocking solution for 2 h at 37 °C. At the same temperature and for the same time 100 µl Goat anti-human-HRP (Meridian Life Science, Inc., G5G16-0482) diluted 1:500 in blocking solution were poured in each well. After every single passage, the plates were washed 5 times with PBS-Tween 20. To reveal the binding, 100 µl of 5 mg-ABTS tablet (Roche Diagnostics) dissolved in 12 ml of sodium citrate 0.05 M, pH 3 and supplemented with 1:1000 dilution hydrogen peroxide (Carlo Erba) were added and after 15, 30, 45 and 60 min the absorbance at 405 nm was measured with a Microplate Reader (Bio-Rad). For the whole measuring time the plates were left in the dark. IC50 analysis of ELISA test was performed with Prism. The test was performed in triplicate.

### Competitive binding in ELISA

Antigen-coated and blocked microplate was prepared as reported above in Materials and Methods ELISA assay. 0.06 µg/ml of H5K1 (concentration of IC_50_) were left incubating for 1 h at 37 °C with serial dilutions of laminarin (from 400 to 0.000045 µg/ml) in blocking solution. Then, the serial dilution solution of laminarin and antibody were added to the plate and left for 1 h at 37 °C. The plate was washed and 100 µl/well of Goat anti-human-HRP (Meridian Life Science, Inc., G5G16-0482) diluted 1:500 in blocking solution was added to the plate and incubated for 1 h at 37 °C. After washing the binding was revealed with 100 µl/well of 5 mg-ABTS tablet (Roche Diagnostics) dissolved in 12 ml of sodium citrate 0.05 M, pH 3 and supplemented with 1:1000 dilution hydrogen peroxide (Carlo Erba). The absorbance was read at 405 nm after 15, 30, 45 and 60 min.

### Flow cytometry and immunofluorescence

From an overnight inoculum, microorganism cells or conidia were washed with RPMI + MOPS (0.165 M, pH 7) and 3.0 × 10^6^ cells were pelleted, resuspended in Phosphate buffered saline (PBS) containing 3% (w/v) BSA (Bovine Serum Albumin Sigma Aldrich) and put in contact with 12 µg/ml of the H5K1 for 1 h at room temperature. The cells and the conidia were washed with PBS and marked with anti-human IgG FITC antibody (Abcam, ab97224) 1:150 in PBS + 3% BSA for 1 h. The cells and conidia were washed and fixed with paraformaldehyde (Carlo Erba) 4% in PBS 1 h at 4 °C. After fixing and washing with PBS, the pellet was resuspended in 400 µl of PBS and splitted in two tubes. The samples of *Candida auris* were analysed respectively using flow cytometry and immunofluorescence while the others just in immunofluorescence^47^. A Flow cytometer (FACScanto II, BDBioscences, Erembodegem, Belgium), equipped with three lasers (488 nm, 633 nm, 405 nm) was employed to collect and quantitate FITC fluorescence from different samples. Both autofluorescence and fluorescence derived from aspecific binding of FITC-conjugated secondary Ab were quantitated by flow cytometry.

### Growth inhibition assay

The growth inhibitory activity of H5K1 was tested as reported by Magliani^48^, with some modifications. In brief, 150–250 cells of *C. auris* in 10 µl of PBS were incubated with 100 µl of hmAb at 250, 100 and 50 µg/ml (each concentration was tested in triplicate) and incubated for 18 h at 37 °C. The inhibition was evaluated by seeding the yeast on Potato Dextrose Agar (PDA) ((Sigma-Aldrich) plates. The plates were incubated at 37 °C for 48 h and the inhibition was calculated by count of the CFU. The assay was performed in triplicate in three different days^25,40^.

### Adhesion assay

In order to investigate a potential protective effect of H5K1 in preventing fungal adhesion to human cells, *C. auris* cells were left adhering to a monolayer of HeLa cells together with the humanized mAb 2G8. 1.0 × 10^4^ cervical cancer cells HeLa were resuspended in RPMI + *10% FBS (pH 7) and plated in a 96-well plate for 2 h at 37 °C + 5% CO_2_. After incubation the cells not yet attached at the bottom of the wells were washed away with RPMI + MOPS (0.165 M, pH 7) and 1.0 × 10^4^ cells of *C. auris* resuspended in RPMI + MOPS (0.165 M, pH 7) were put on to reach a 1:1 ratio HeLa:yeast cells. Together with the yeast, 12 µg/ml of the humanized mAb 2G8 were added. PBS pH 7 was used in the control. The plate was left at 37 °C for 1 h and after washing 5 times, HeLa cells were lysed with PBS 0.1% Triton X-100 pH 7 for 15 min. RT. The suspension was plated in PDA plate and incubated at 37 °C for 48 h. This experiment has been repeated two times in triplicate^40,49^.

### Monocytes preparation

Cultures of macrophages were prepared from leukocyte buffy coats obtained from healthy donors. Briefly, the peripheral blood mononuclear cells were isolated by Lymphoprep density gradient medium (Stemcell Technologies) and monocytes were separated from lymphocytes by adherence to plastic dishes. Monocytes were cultured in RPMI containing 10% foetal calf serum and 1% antibiotics at 37 °C in a 5% CO_2_ atmosphere for 10–12 days, at which time the monocytes had matured into macrophages and formed a monolayer.

### Phagocytosis assay

From an overnight inoculum, *C. auris* cells were washed with RPMI + MOPS (0.165 M, pH 7) and labelled with 1 mg/ml Fluorescein-5-isothiocyanate (FITC, Sigma-Aldrich) in PBS for 10 min RT. After washing 250 µg/ml of H5K1 were left interacting for 1 h at 37 °C. Meanwhile macrophages were treated with 100 nM LysoTracker Red (Thermo Fisher Scientific, Waltham, MA, USA) for 45 min and washed with PBS. Yeasts and macrophages were counted in order to have a final ratio *C. auris* cells:macrophages 1:1. They were left 15, 30 and 180 min at 37 °C and then washed and fixed with paraformaldehyde (Carlo Erba) 4% in PBS 20 min at 4 °C. After washing again, the cells were scraped gently and acquired by flow cytometry. 15,000 cell events were acquired for each sample^50^.

### MIC assays

To evaluate the susceptibility of *C*. *auris* to CAS and AMB and their combination with H5K1, we followed the EUCAST antifungal MIC microdilution method^51^. Antifungal drugs were purchased from Sigma-Aldrich. CAS and AMB concentration ranges analysed were reported in EUCAST document. Those concentrations were tested alone and in combination with 0.25, 2.5, 25 and 250 µg/ml of humanized antibody. The wells with 100 µl of 2X final antifungal drugs concentration in RPMI 2% G medium were inoculated with 100 µl (1–5 × 10^5^ CFU/ml) of yeast suspension of *C. auris*. The monoclonal antibody was added to the samples of yeast suspension to test the drug-antibody combination. The plates were incubated at 37 °C for 24 and 48 h and read after incubation at 405 nm with a Microplate Reader (Bio-Rad). With the term MIC50 we consider the lowest concentration that inhibits the 50% of the growth compared to drug-free control and with MIC90, the lowest concentration that inhibits the 90% of the growth compared to drug-free control. The assays were performed three times in triplicate.

### Time-kill curve assays

Based on MIC results a restricted range of drug concentration was used (4, 2, 1, 0.5, 0.25, 0.125 and 0.0625 µg/ml for caspofungin and 4, 2, 1, 0.5, 0.25, 0.125, 0.0625 and 0.03125 µg/ml for amphotericin B). In each well of a 96-well plate, 100 µl of 2X final antifungal drugs concentration in RPMI 2% G medium and 100 µl containing 1–5 × 10^5^ CFU/ml of *C. auris* suspension were put together. 250 µg/ml of humanized antibody was added to see the effect of the combination. 5 mM phosphate buffer was used as control. The plates were incubated at 37 °C for 0, 2, 4, 6, 8, 24 and 48 h. The samples were spread (tenfold dilutions were used when necessary) in PDA plates and left at 37 °C for 48 h before the CFU counting. Time-kills for strain-drug combination were performed a single time.

### Statistical analysis

Data were plotted and analysed by GraphPad Prism 8 software. The statistical significance in adhesion and phagocytosis assays were assessed through a two-tailed Student’s *t* test while in growth inhibition results, the statistical significance was assessed by One-way Anova test.

## Supplementary Information

Supplementary Information 1.

Supplementary Video 1.

Supplementary Video 2.

## Data Availability

All data generated and analyzed during this study are included in this published article (and its Supplementary Information files) and available from the corresponding author on reasonable request.
