# Supplementary material for: A new humanized antibody is effective against pathogenic fungi in vitro
Source: Sci Rep. 2021 Sep 30;11:19500. doi: 10.1038/s41598-021-98659-5 (PMC8484667; doi:10.1038/s41598-021-98659-5)
Supplement: Supplementary file 1 — Supplementary Information 1. [file 41598_2021_98659_MOESM1_ESM.pdf]

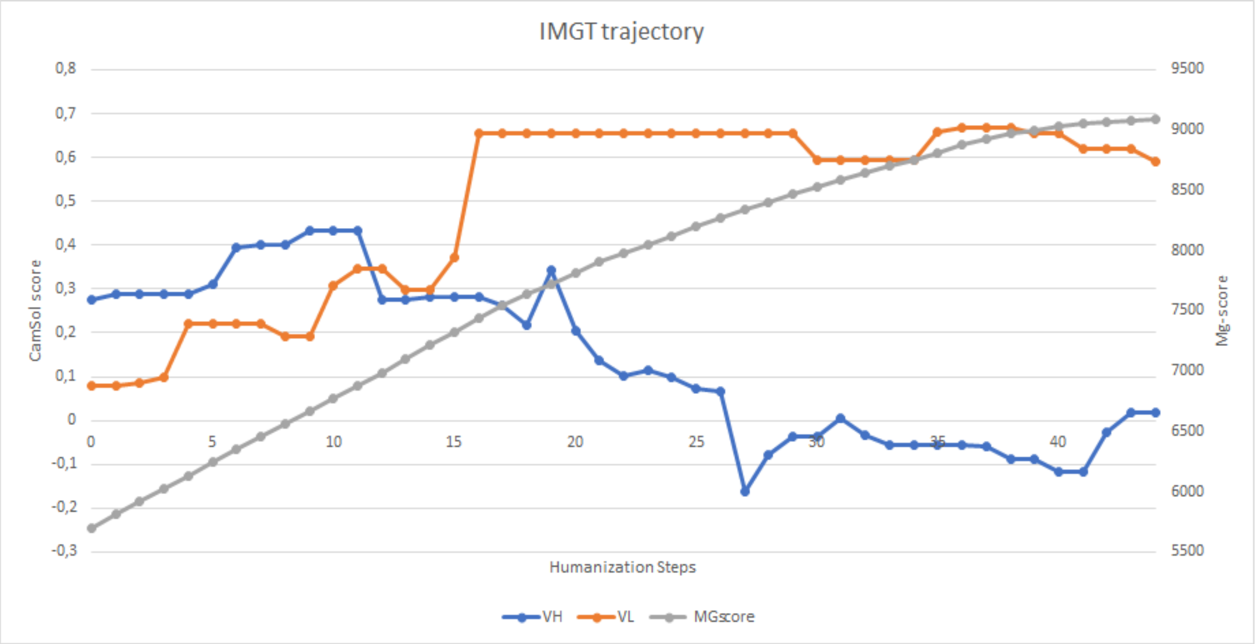

Figure S1 (supplementary information): CamSol Intrinsic solubility scores (mayor y-axes) and humanization MG-score (minor y-axes) for the humanization trajectories in sequence space starting from the murine sequence (step 0): the results for the second protocol described in Methods, upon fixing the CDR residues corresponding to the IMGT numbering. High CamSol score corresponds to high solubility; MG-scores above 6383 are classified as human.

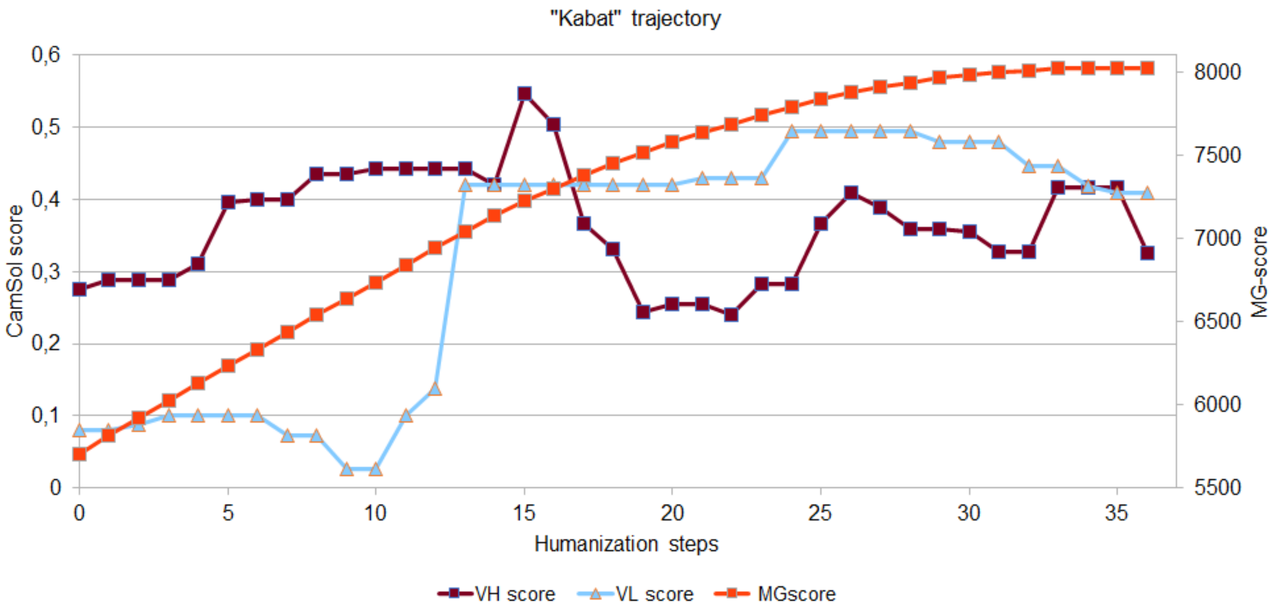

Figures S2 (supplementary information): CamSol Intrinsic solubility scores (mayor y-axes) and humanization MG-score (minor y-axes) for the humanization trajectories in sequence space starting from the murine sequence (step 0): the results for the third protocol, upon fixing the CDR residues corresponding to the Kabat numbering. High CamSol score corresponds to high solubility; MG-scores above 6383 are classified as human.

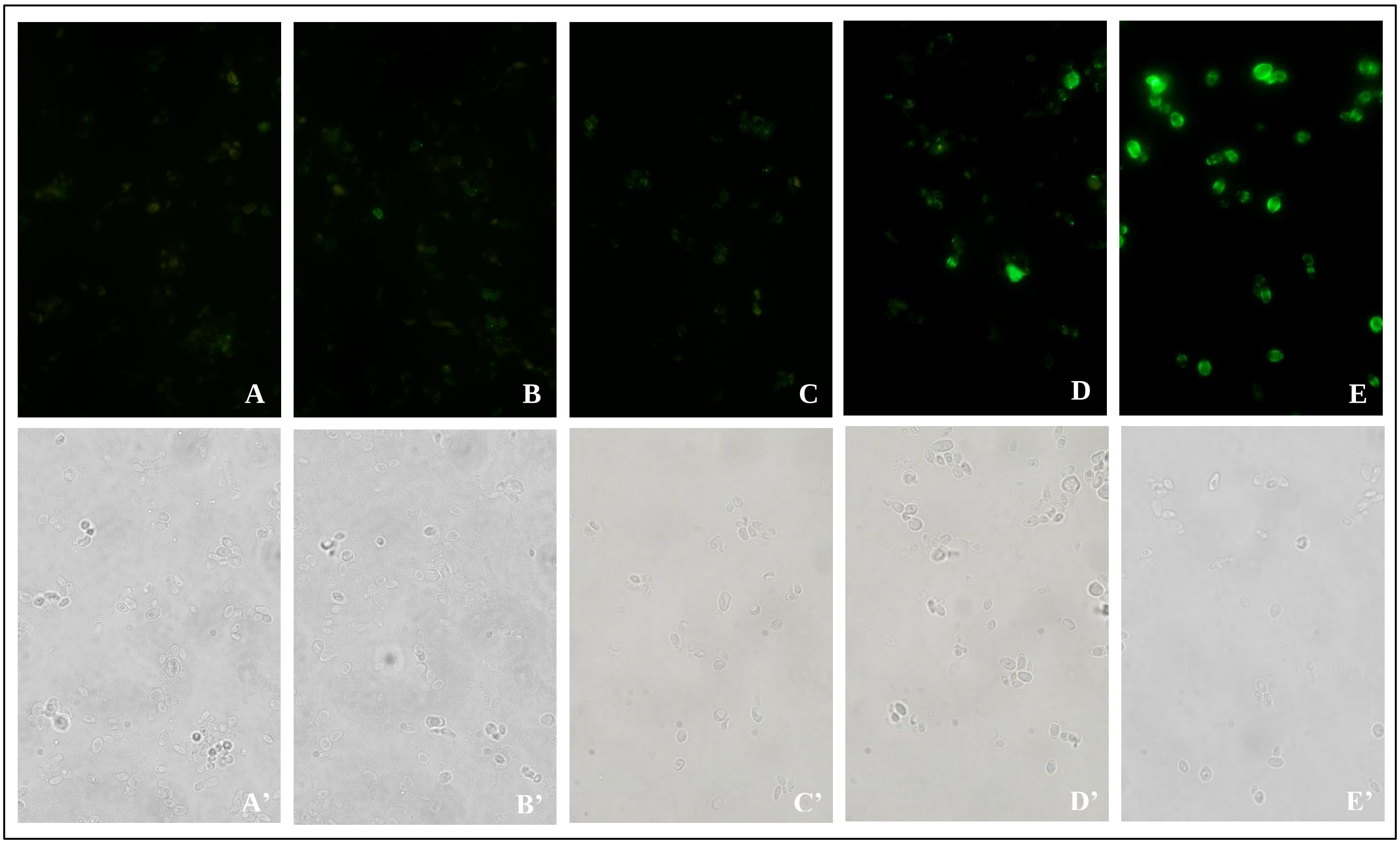

Figure S3 (supplementary information): competitive binding of H5K1 to *C. auris* cells after treatment with different concentrations of laminarin. PBS pre-treatment was used as control. Considering that the humanized antibody is bivalent, and that more than one antibody can bind a single laminarin polysaccharide, the ratios laminarin:H5K1 in μg/ml were respectively: A-A’ 40:1, B-B’ 16:1, C-C’ 4:1, D-D’ 1:1, E-E’ 0:1.

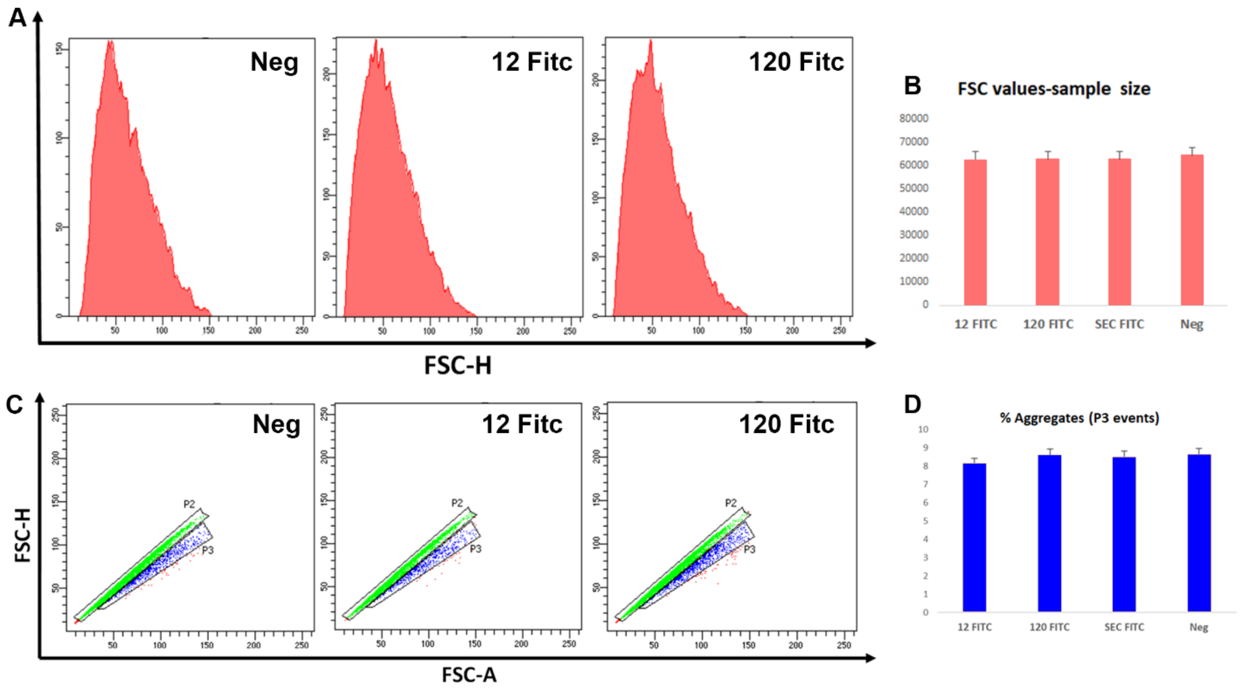

Figure S4 (supplementary information): flow cytometry is also suited for measurements of cell size, interaction, aggregation or shape using non-labelled cells by means of analysing their light scattering characteristics. Yeast aggregation is not induced by antibody labelling, as demonstrated by flow cytometric detection of both total event size (FSC histogram (A-B)) and dimensional aggregates (dot plot FSC-A vs FSC-H (C-D)) Data were collected as FSC values and percentages of aggregates.

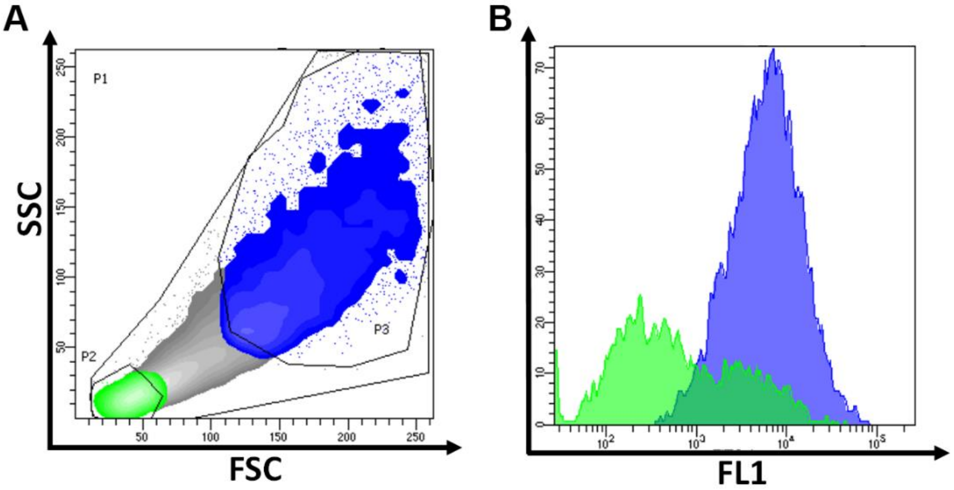

Figure S5 (supplementary information): Fluorescence Intensity highlights a differential labelling of the antibody on different subpopulations of *Candida albicans* during hyphal transition. Green events represent small cells, putative scarce residual yeasts, partially germinating [51], whereas blue events represent hyphal morphogenetic state (hyphal aggregates, scarcely represented rare excluded from the analysis) (A). Hystogram (B) overlays puts in light a higher Mean Fluorescence Intensity (MFI) on blue events in respect to the green ones, in agreement with the higher presence of 1,3 Beta-glucan in hyphal state, as reported [24].

VID. 1A: binding analysis by means of immunofluorescence technique – Control FITC

VID. 1B: binding analysis by means of immunofluorescence technique – hmAb H5K1 FITC
